# *In silico* analysis suggests interaction between Ebola virus and the extracellular matrix

**DOI:** 10.3389/fmicb.2015.00135

**Published:** 2015-02-19

**Authors:** Veljko Veljkovic, Sanja Glisic, Claude P. Muller, Matthew Scotch, Donald R. Branch, Vladimir R. Perovic, Milan Sencanski, Nevena Veljkovic, Alfonso Colombatti

**Affiliations:** ^1^Center for Multidisciplinary Research, Institute of Nuclear Sciences VINCA, University of BelgradeBelgrade, Serbia; ^2^Luxembourg Institute of Health (former Centre de Recherche Public de la Santé)/Laboratoire National de SantéLuxembourg, Luxembourg; ^3^Department of Biomedical Informatics, Arizona State UniversityScottsdale, AZ, USA; ^4^Center for Environmental Security, Biodesign Institute and Security and Defense Systems Initiative, Arizona State UniversityTempe, AZ, USA; ^5^Canadian Blood Services, Center for InnovationToronto, ON, Canada; ^6^Innovation Center of the Faculty of Chemistry, University of BelgradeBelgrade, Serbia; ^7^Divisione di Oncologia Sperimentale, Centro di Riferimento Oncologico CRO-IRCCSAviano, Italy

**Keywords:** Ebola virus, glycoprotein GP, endothelial extracellular matrix, EMILINs, *in silico* analysis

## Abstract

The worst Ebola virus (EV) outbreak in history has hit Liberia, Sierra Leone and Guinea hardest and the trend lines in this crisis are grave, and now represents a global public health threat concern. Limited therapeutic and/or prophylactic options are available for people suffering from Ebola virus disease (EVD) and further complicate the situation. Previous studies suggested that the EV glycoprotein (GP) is the main determinant causing structural damage of endothelial cells that triggers the hemorrhagic diathesis, but molecular mechanisms underlying this phenomenon remains elusive. Using the informational spectrum method (ISM), a virtual spectroscopy method for analysis of the protein-protein interactions, the interaction of GP with endothelial extracellular matrix (ECM) was investigated. Presented results of this *in silico* study suggest that Elastin Microfibril Interface Located Proteins (EMILINs) are involved in interaction between GP and ECM. This finding could contribute to a better understanding of EV/endothelium interaction and its role in pathogenesis, prevention and therapy of EVD.

## Introduction

Ebola virus (EBOV) is an aggressive pathogen that causes a highly lethal hemorrhagic fever syndrome in humans and nonhuman primates with mortality rates ranging from 50 to 90% (Peters and Khan, 1999). EBOV belongs to the *Filoviridae*, order *Mononehavirales*. The members of this genus of ssRNA viruses are called ebolaviruses and include five known virus species: Bundibugyo virus (BDBV), Sudan virus (SUDV), Taï Forest virus (TAFV), Reston virus (REBOV) and one called simply Ebola virus (EBOV, formerly Zaire Ebola virus) which caused a number of outbreaks during the past 40 years. The current outbreak began in December 2013 in Guinea and spread into Liberia, Sierra Leone and Nigeria, and is the largest known EV disease (EVD) outbreak; that, at the beginning was not adequately controlled and now represents a global public health threat concern (Gire et al., 2014). In our previous work, we studied the phylogeography of EBOV and found the origin of EBOV 2014 to be strains from 1976 in Zaire. This origin ultimately seeded a basal clade that became extinct while also maintaining a 25-year lineage in Gabon. We also found that the Guinea and Sierra Leone viruses are descendants of an earlier outbreak in Gabon that also spread to Democratic Republic Congo (Gire et al., 2014).

Disseminated intravascular coagulation (DIC) is a prominent manifestation of EBOV infection (Baskerville et al., 1985). DIC is a syndrome characterized by coagulation abnormalities including systemic intravascular activation of coagulation leading to widespread deposition of fibrin in the circulation. It is imperative to understand the molecular mechanisms underlying this phenomenon and other processes involved in the pathogenesis of the disease in order to develop an efficient therapy and/or vaccine. Despite extensive research on EVD, many questions concerning the virus host interaction of EBOV remain open. This also concerns the important question of the role of endothelial cells in the pathogenesis of EBOV hemorrhagic fever (HF). The EBOV infection of endothelial cells has been well documented in nonhuman primates (Baskerville et al., 1985; Geisbert et al., 1992; Jaax et al., 1996; Davis et al., 1997; Ryabchikova et al., 1999). It was shown that plasma or serum from convalescing patients enhanced this infection and that this enhancement was mediated by antibodies to the viral glycoprotein (GP) and by the complement component C1q (Takada et al., 2001, 2003). Previous studies suggested that the EBOV glycoprotein (GP) is the main determinant of vascular cell injury and leads to direct structural damage of endothelial cells that triggers the hemorrhagic diathesis (Yang et al., 1998, 2000). It has been shown that abnormalities associated with EBOV HF are not directly caused by cytolysis of endothelial cells but rather by an indirect mechanism (Geisbert et al., 2003).

Previously, we studied the interactions between the anthrax protective antigen (PA) from *Bacillus anthraces* and target host proteins by *in silico* applying the informational spectrum method (ISM) and by *in vitro* assays (Doliana et al., 2008). Our findings suggested that the PA interaction with the cell surface receptor is not sufficient to explain the vascular lesions and prominent hemorrhages caused by *Bacillus anthracis*. We proposed a role for the vascular associated proteins such as EMILINs (Elastin Microfibril Interface Located Protein) which could function as potential “decoy receptor binding proteins” (Doliana et al., 2008).

Here we investigated possible interaction between EBOV GP and EMILINs using the ISM technique. Results of this *in silico* study suggest that EMILINs are involved in interaction between EBOV and the endothelial extracellular matrix (ECM). We discuss implications of possible GP/EMILIN interaction in EVD pathogenesis, prevention and therapy.

## Materials and methods

### Virus sequences

Sixteen non-redundant GP1 sequences from Ebola virus collected during outbreaks from 1976 to 2014 have been investigated (Data Sheet 1) and 101 GP1 sequences from the EBOV outbreak 2014 (Data Sheet 2). All sequences were taken from GenBank and UniProt databases and their accession numbers are given in Data Sheet 1 and Data Sheet 2.

### Electron-ion interaction potential (EIIP)

The intermolecular interactions in biological systems encompass two basic steps, (i) specific long-distance targeting of interacting molecules and (ii) chemical bond formation between interacting molecules. The first step is determined by selective long-range forces which are efficient at a distance longer than one linear dimension of the interacting macromolecules (10^2^-10^3^ Å′) (Fröhlich, 1968, 1970, 1975). These forces directly influence number of productive collisions between interacting molecules. Before chemical bond formation take place, reacting molecular regions must be positioned close enough (at a distance of ≈2 Å) and the appropriate atoms must be held in the correct orientation for the reaction that is to follow, because the attractive forces involved in the recognition and binding of molecules include all the weak non-covalent forces (van der Waals, hydrogen bonding, ionic interactions, etc.). For this reason, stereochemical complementarity between interacting molecules is essential for the second step.

It has been proposed that the number of valence electrons and the electron-ion interaction potential (EIIP) representing the main energy term of valence electrons are essential physical parameters determining of the long-range properties of biological molecules (Veljkovic, 1980). We showed (Veljkovic and Lalovic, 1976; Veljkovic, 1980) that EIIP can be determined for organic molecules by the following simple equation derived from the “general model pseudopotential” (Veljkovic and Slavic, 1972; Veljkovic, 1973; Veljkovic and Lalovic, 1973),

(1) $$W=0.25\frac{Z^{*}\sin(1.04\pi Z^{*})}{2\pi }$$

where Z^*^ is the average quasivalence number (AQVN) determined by

(2) $$Z^{*}=\frac{1}{N}\sum_{i\;=\;1}^{m}n_{i}Z_{i}$$

where Z_*i*_ is the valence number of the *i*-th atomic component, *n_i_* is the number of atoms of the *i*-th component, *m* is the number of atomic components in the molecule, and Nis the total number of atoms. The EIIP values calculated according to Equations (1) and (2) are given in Rydberg (Ry).

### Informational spectrum method (ISM)

The informational spectrum method (ISM) technique (Veljkovic et al., 1985; Veljkovic and Cosic, 1987; Lazovic, 1996; Cosic, 1997) is based on a model of the primary structure of a protein using a sequence of numbers, by assigning to each amino acid the corresponding value of EIIP (Table 1). The obtained numerical sequence, is subjected to a discrete Fourier transformation which is defined as follows,

(3) $$X(n)=\sum_{m\;=\;1}^{N}x(m)e^{-i2\pi n(m-1)/N},n=1,2,\ldots ,N/2$$

where x(m) is the m-th member of a given numerical series, N is the total number of points in this series, and X(n) are discrete Fourier transformation coefficients. These coefficients describe the amplitude, phase and frequency of sinusoids, which comprised the original signal. The absolute value of complex discrete Fourier transformation defines the amplitude spectrum and the phase spectrum. The complete information about the original sequence is contained in both spectral functions. However, in the case of protein analysis, relevant information is presented in an energy density spectrum (Veljkovic et al., 1985), which is defined as follows,

(4) $$S(n)=X(n)X^{*}(n)=|X(n){|}^{2},n=1,2,\ldots ,N/2$$

**Table 1:** **The electron-ion interaction potential (EIIP) used to encode amino acids**.

**Amino acid**	**EIIP [Ry]**
Leu	0.0000
Ile	0.0000
Asn	0.0036
Gly	0.0050
Glu	0.0057
Val	0.0058
Pro	0.0198
His	0.0242
Lys	0.0371
Ala	0.0373
Tyr	0.0516
Trp	0.0548
Gln	0.0761
Met	0.0823
Ser	0.0829
Cys	0.0829
Thr	0.0941
Phe	0.0946
Arg	0.0959
Asp	0.1263

**Figure 1:**
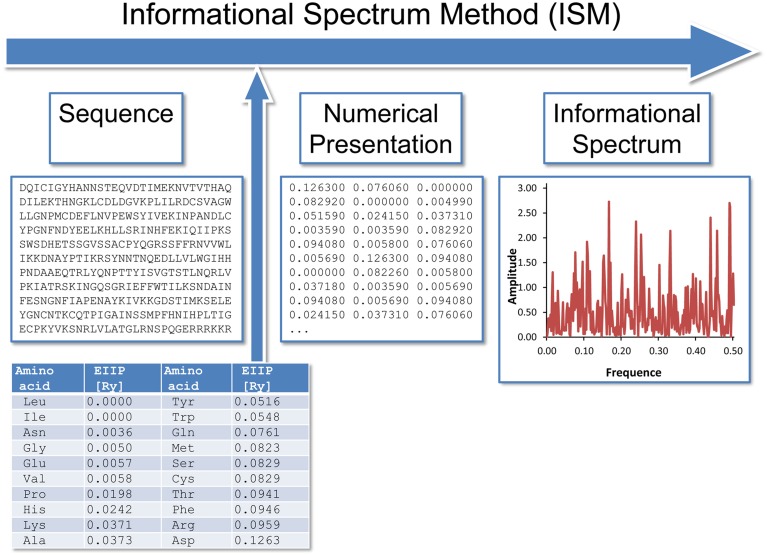
**The schematic presentation of the ISM**.

In this way, sequences are analyzed as discrete signals. It is assumed that their points are equidistant with the distance *d* = 1. The maximal frequency in a spectrum defined in this way is *F* = 1/2 *d* = 0.5. The frequency range is independent of the total number of points in the sequence. The total number of points in a sequence influences only resolution of the spectrum. The resolution of the N-point sequence is 1/n. The n-th point in the spectral function corresponds to a frequency *f*_(n)_ = nf = n/N. Thus, the initial information defined by the sequence of amino acids can now be presented in the form of the informational spectrum (IS), representing the series of frequencies and their amplitudes. The schematic presentation of the ISM is given in Figure 1.

The IS frequencies correspond to the distribution of structural motifs with defined physico-chemical characteristics responsible for biological function of a protein. When comparing proteins which share the same biological or biochemical function, the ISM technique allows detection of code/frequency pairs which are specific for their common biological properties, or which correlate with their specific interaction. This common informational characteristics of sequences is determined by cross-spectrum or consensus informational spectrum (CIS). A CIS of N spectra is obtained by the following equation,

(5) $$C(j)=\prod_{i\;=\;1}^{N}S(i,j)$$

where S(i,j) is the j-th element of the i-th power spectrum and C(j) is the j-th element of CIS. Thus, CIS is the Fourier transform of the correlation function for the spectrum. In this way, any spectral component (frequency) not present in all compared ISs is eliminated. Peak frequencies in CIS are common frequency components for the analyzed sequences. A measure of similarity for each peak is a signal-to-noise ratio (S/N), which represents a ratio between signal intensity at one particular frequency and the main value of the whole spectrum. If one calculates a CIS for a two or more of proteins, which have different primary structures, and finds strictly defined peak frequencies, it means that the analyzed proteins participate in mutual interactions or have a common biological function. The characteristic frequencies are, up to now, obtained for the more than 20 groups of proteins (oncogenes, kinases, interferons, growth factors, haemoglobins, etc.) as well as for several types of DNA regulatory sequences (promotors, terminators, enhancers, SOS-operators) (Cosic, 1997).

The ISM was successfully applied in structure-function analysis of different protein sequences, as well as in *de novo* design of biologically active peptides (Huang et al., 2005; Veljkovic et al., 2008, 2009a,b, 2014; Tintori et al., 2010; Pirogova et al., 2011, 2012; Glisic et al., 2012; Nwankwo, 2013; Srdic-Rajic et al., 2013; Deng and Huang, 2014).

### GP1 receptor modeling

The full sequence of GP1 was retrieved from GenBank, accesion number: AIE11800 and modeled on Phyre 2 server (Protein Homology/analogY Recognition Engine V 2.0) (Kelley and Sternberg, 2009). The sequence is automatically searched for homologs, and if found, the 3D coordinates of model are built according to known crystal structures. The sequence was recognized and aligned with the crystal structure of Ebola virus glycoprotein, PDBID 3CSY (Lee et al., 2008). Known regions were modeled according to known coordinates of the crystal structure, while the remaining regions were ab initio modeled. The modeling was carried out under intensive mode. The confidence in the model was 47% for 220 and >90% accuracy.

### Ligand optimization

Ligands were built in VEGA ZZ (Pedretti et al., 2004), protonated according to physiological conditions and optimized on PM6 level of theory using MOPAC 2009.

### Molecular docking

Ligand and receptor were prepared using AutoDock Tools 1.5.7. (Sanner, 1999; Morris et al., 2009). The docking was carried with Autodock Vina 1.1.2. (Trott and Olson, 2010) The whole receptor conformational space was searched, using grid box dimensions 60 × 60 × 60 Å^3^. After selection of conformations that were docked near recognized sequences important for binding of anti-malarials, the binding pocket was recognized, grid box centerd to occupy close amino acid residues and set to dimensions of 20 × 20 × 20 Å^3^. The docking was carried out with and without weighting of hydrophilic interactions. In case of hydrophilic weighting, the value was set to −1.20 (compared to default weight_hydrogen = −0.587439 value). The exhaustiveness was set to 250.

## Results

**Figure 2:**
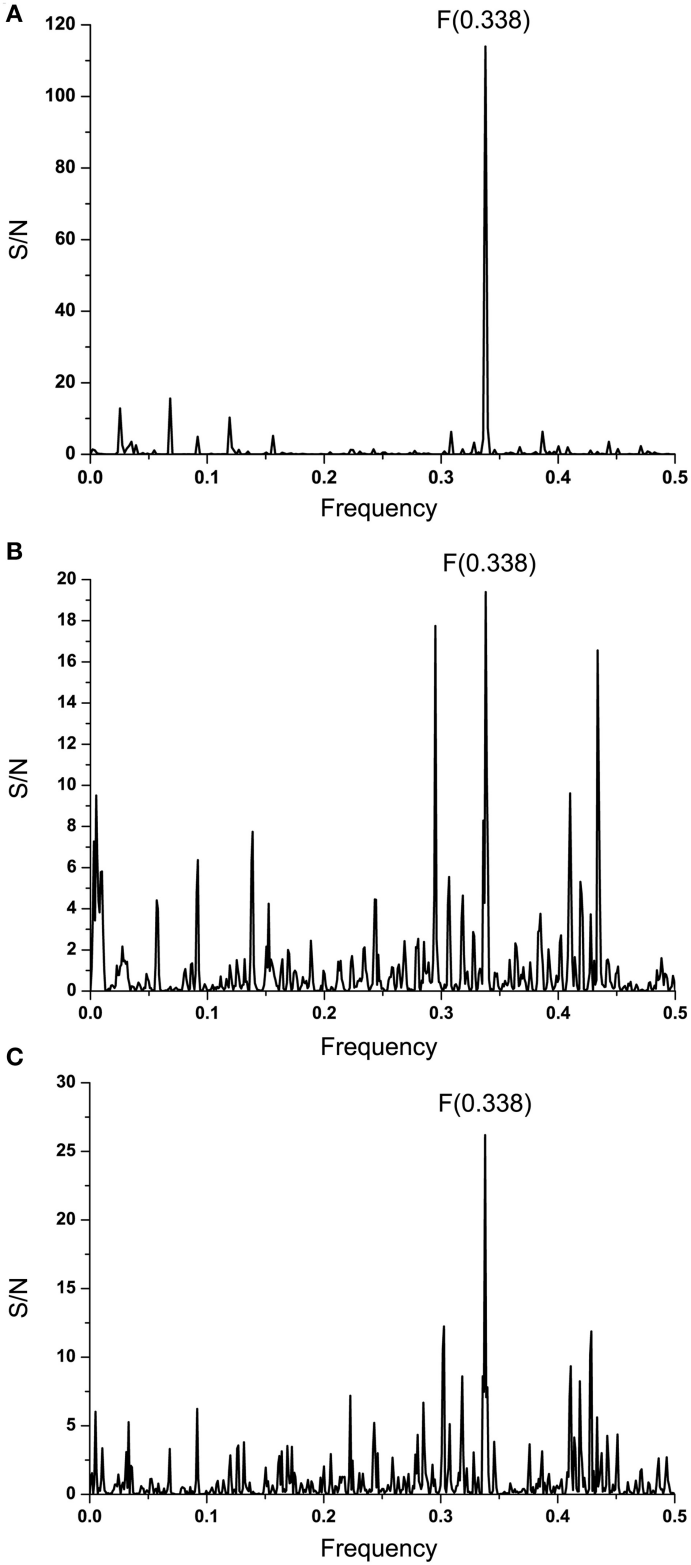
**Cross-spectral analysis of EBOV-2014 GP1, EMILINs and C1q. (A)** CS of C1q and EBOV-2014; **(B)** CS of C1q and EMILIN-1, EMILIN-2 and EMILIN-3; **(C)** CS of EBOV-2014 GP1 and EMILIN-1, EMILIN-2 and EMILIN-3.

Complement component C1q and Ebola virus GP1 are involved in the antibody-dependent enhancement (ADE) of the infection (Takada et al., 2001, 2003). Previously, we showed that proteins that are recognized by the same antibody encode for common information determining the long-range recognition and targeting (interaction on distances >5 Å) of this antibody (Veljkovic et al., 2003; Vergara-Alert et al., 2012). This information is represented by frequencie(s) in the informational cross-spectrum (CS) of immunologicaly crossreactive proteins. Efficacy of recognition and targeting of crossreactive proteins by the antibody is determined with the amplitude and S/N values corresponding to the common frequency. To identify the common information encoded by EBOV GP1 and C1q, ISM analysis was performed on all non-redundant EBOV-2014 GP1 amino acid sequences in GenBank (Data Sheet 2). Figure 2A shows CS of C1q and GP1 of EBOV strains KM233035, representing 99 of 101 GP1 homologous sequences from the outbreak 2014 in GenBank (Data Sheet 2), as well as KJ660346 and KJ660348. This CS is characterized by a single peak corresponding to the IS frequency *F*_(0.338)_, which represents the common information encoded by primary structures of GP1 and C1q. This result suggests possible crossreactivity between these proteins, but also their direct mutual interaction.

**Figure 3:**
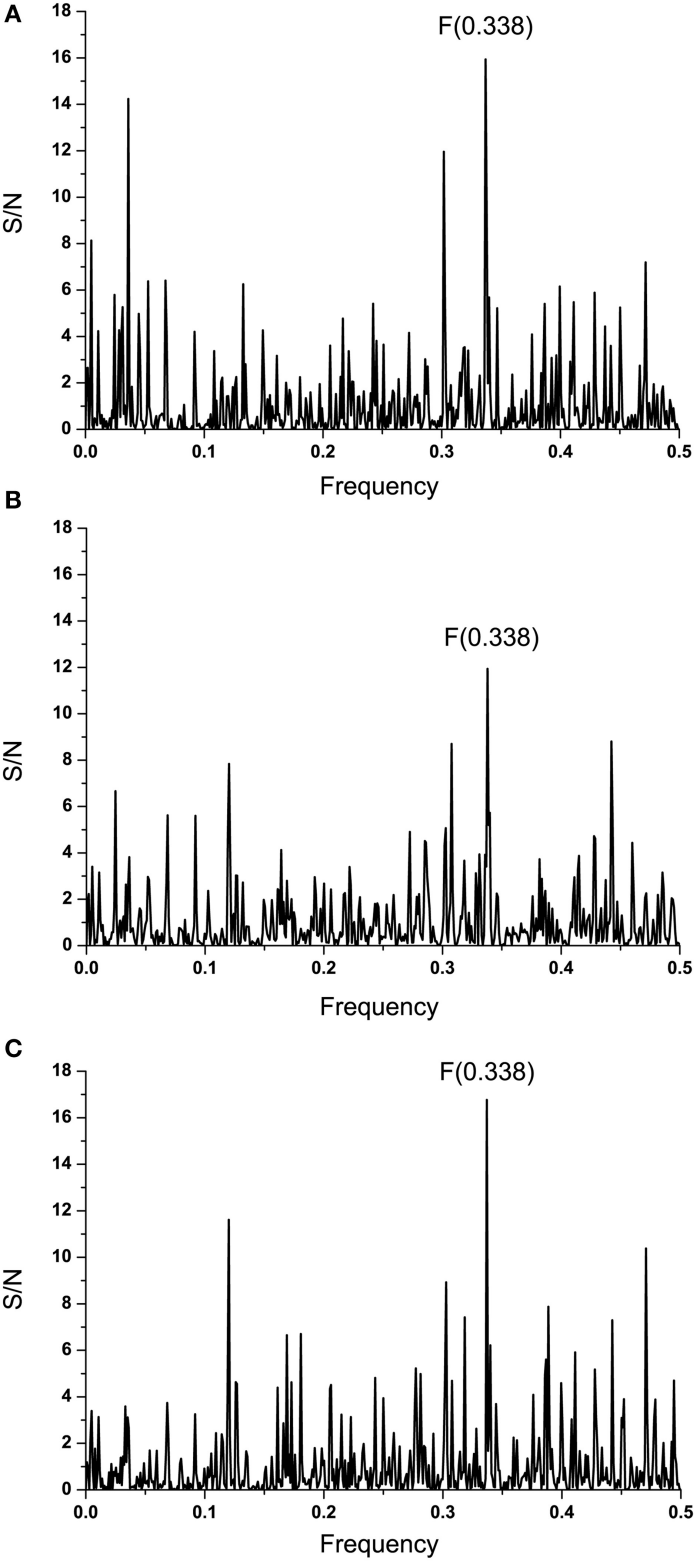
**Cross-spectra of EMILINs and GP1 from EBOV-2014 KM233035. (A)** CS of EMILIN-1 and GP1 KM233035; **(B)** CS of EMILIN-2 and GP1 KM233035; **(C)** CS of EMILIN-3 and GP1 KM233035.

It has been reported that elastin microfibrillar interface proteins (EMILINs), which are predominantly expressed in the ECM, share the C-terminal gC1q domain typical of the gC1q/TNFsuperfamily members (Colombatti et al., 2012). The CS of C1qc, representing the major constituent of the human complement subcomponent C1q, and of EMILINs shows that these proteins share dominant information which is represented by the IS frequency *F*_(0.338)_ (Figure 2B). The CS of EBOV-2014 GP1 (KM233035) and EMILINs contains the dominant peak at the frequency *F*_(0.338)_, representing the common information encoded by these viral and human proteins (Figure 2C). The prominent peaks in CSs of EBOV GP1 (KM233035) and EMILIN-1, EMILIN-2 and EMILIN-3 are also at frequency *F*_(0.338)_ (Figure 3). These results of Figures 2, 3 suggest putative direct interaction or immunological cross-reactivity between EBOV GP1 and EMILINs.

**Figure 4:**
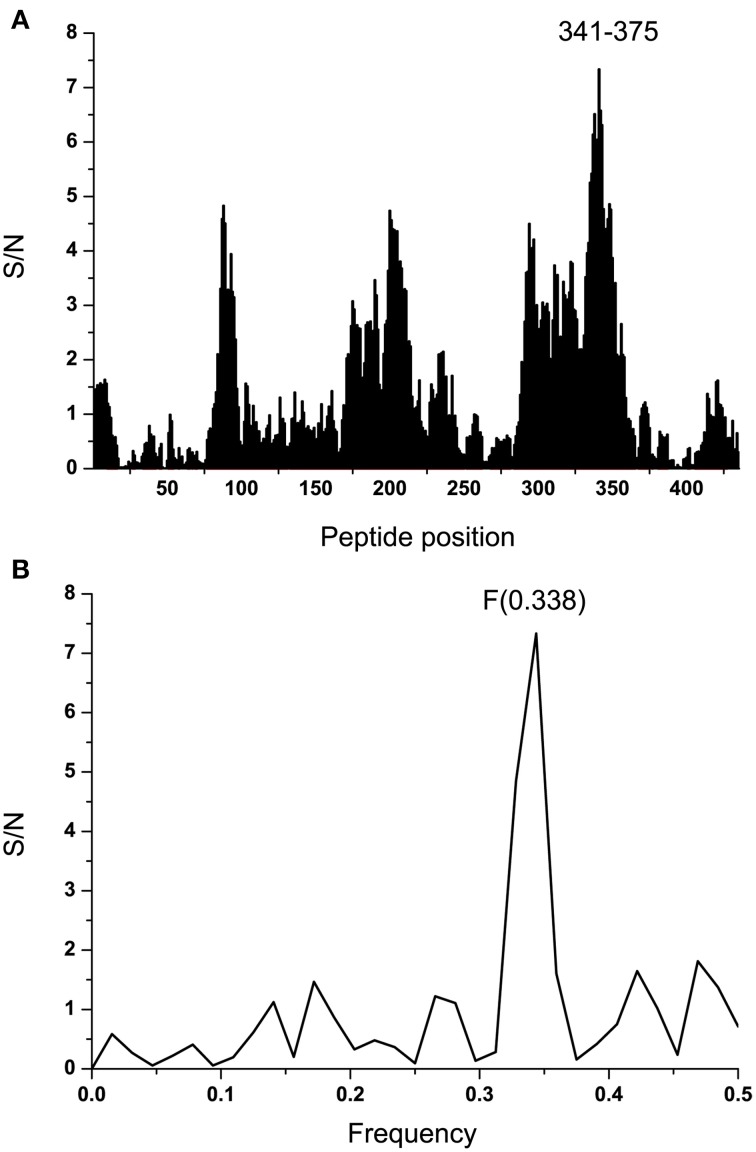
**Mapping of the putative interacting sites of EBOV-2014 (KM233035) GP1 and EMILINs. (A)** Position of the domain VIN_EBOLA1_ in the primary structure of GP1. **(B)** IS of the VIN_EBOLA1_ domain (residues 341–375).

To identify the domain which is essential for information corresponding to the IS frequency *F*_(0.338)_, the computer scanning of the primary structure of EBOV GP1 (KM233035) with peptides of different lengths was performed. This analysis showed that the main contribution to the frequency *F*_(0.338)_ comes primarily from the domain 341-375 (a.a. numbering in maturated protein without 32 residues of the signal peptide) (Figure 4A). According to the ISM concept, this region of GP1 (denoted VIN_EBOV1_) is essential for possible long-range interaction or immunological crossreactivity between GP1 and EMILINs. Previously it was shown that domains of proteins which are essential for their long-range interaction overlap their mutual binding site or that they are located in its vicinity (Veljkovic et al., 2009a,b; Colombatti et al., 2012; Vergara-Alert et al., 2012). Figure 4B shows the IS of VIN_EBOV1_ containing the dominant peak at the frequency *F*_(0.338)_.

**Figure 5:**
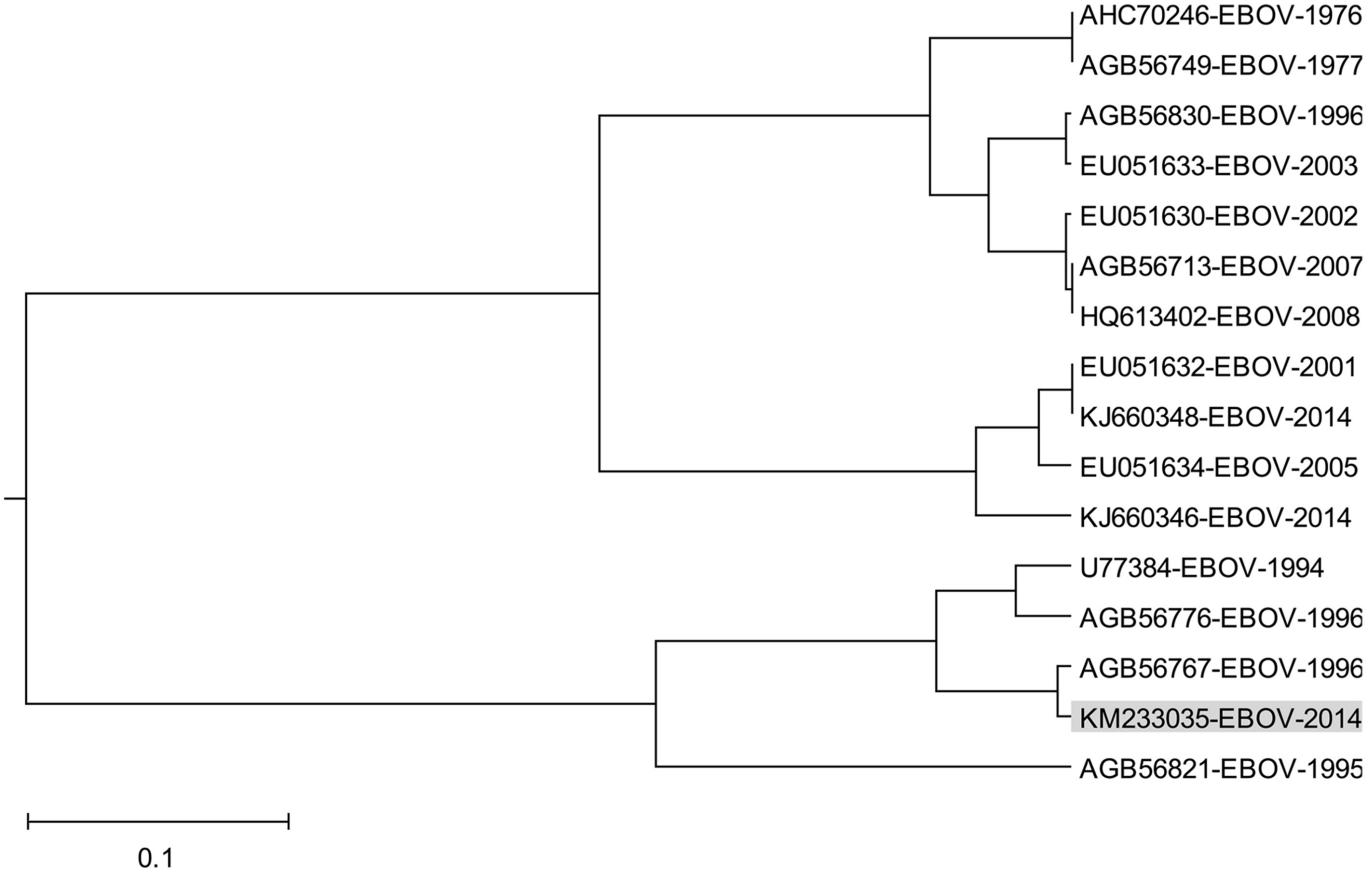
**The ISM-based phylogenetic analysis of non-redundant EBOV GP1**.

**Figure 6:**
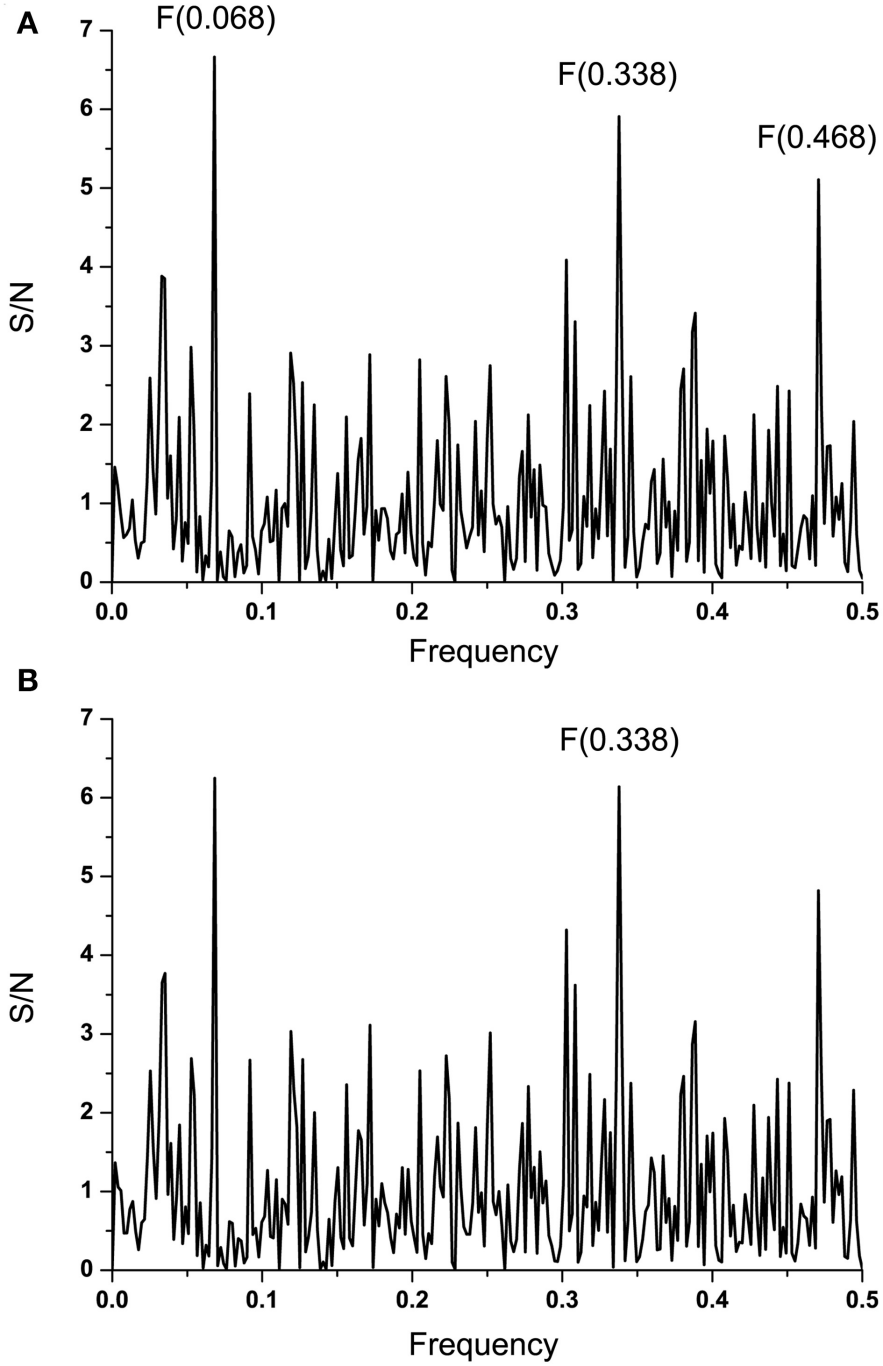
**Effect of the mutation S408G on IS of EBOV-2014 (KM233035) GP1. (A)** IS of GP1 from EBOV-2014 (KM233035). **(B)** IS of EBOV-2014 (KM233035) GP1 with the mutation S408G.

Effect of ADE varies according the Ebola virus species (Takada et al., 2003) indicating that properties of GP1 which correspond to information represented by the IS frequency *F*_(0.338)_ may also differ between species. In order to analyze this variation we performed the ISM-based phylogenetic analysis of all non-redundant sequences of GP1 in GenBank and UniProt databases of Ebola viruses collected between 1976 and 2014 (Data Sheet 1) as described before (Perovic, 2013; Perovic et al., 2013). The amplitude at the frequency *F*_(0.338)_ as an parameter in the ISM-based phylogenetic analysis was used (Perovic et al., 2013) (Figure 5). The group of four viruses collected between 1994 and 1996 and the EBOV strain KM233035, representing the 99 homologous out of 101 GP1 sequences from the current 2014 outbreak in West Africa, form a distinct cluster in this phylogenetic tree. The comparison of IS of GP1 of these viruses shows that these five viruses have significantly higher values of amplitude and S/N at the frequency *F*_(0.338)_ than all other viruses included in Figure 5 (Table 2). This indicates that interaction/crossreactivity of these viruses with C1qc/EMILINs is stronger than in the case of other EBOV. Previously were reported mutations S3459, L346P, S390P, P398L, and S408G of GP1 which differ between GP1 of EBOV-1976 and EBOV-1995 (Rodriguez et al., 1999). These mutations were fixed in all EBOV strains in outbreaks 1994–1996 although they are located within highly variable domain of GP1. The homology analysis of GP1 from EBOV collected during outbreaks from 1994 to 2014 (Data Sheet 3) show that four mutations S3459, L346P, S390P, P398L are the only ones present in 99 out of 101 sequences from EBOV-2014. From this point of view, EBOV-2014 is the most similar to the EBOV from outbreaks 1994–1996. Interestingly, the mutation P398L was not present in any of the GP1 proteins from viruses isolated after 1996 but it re-appeared in EBOV-2014. To assess the potential biological effect of this mutation we introduced the reverse mutation L398P into GP1 of EBOV KM233035. This change resulted in a decrease of the amplitude at the frequency *F*_(0.338)_. This reverse mutation also significantly decreased the amplitudes at two other frequencies *F*_(0.068)_ and *F*_(0.468)_ which we previously reported as important for virus/receptor interaction (Veljkovic et al., submitted). These *in silico* results strongly suggested the biological significance of the mutation P398L which probably modulates interaction between GP1 and host proteins. Finally we investigated the effect of the mutation S408G which disappeared after 1996. Introduction of this mutation into GP1 from EBOV KM233035 resulted in a significant change of the IS and increased the amplitude of the frequency *F*_(0.338)_ (Figure 6). This indicates that acquisition of the mutation S408G by EBOV-2014 could increase the interaction/crossreactivity of these viruses with C1qc/EMILINs.

**Table 2:** **The amplitude and S/N values at the frequency *F*_(0.338)_ in IS of GP1 from EBOV collected 1976-2014**.

**EBOV (Access. No.)**	**Year**	**Amplitude**	**S/N**
AHC70246	1976	3.6	4.6
AGB56749	1977	3.6	4.6
U77384	1994	4.6	5.8
AGB56821	1995	4.9	6.2
AGB56767	1996	4.6	5.9
AGB56776	1996	4.5	5.8
AGB56830	1996	3.8	4.8
EU051632	2001	4.1	5.2
EU051630	2002	3.7	4.8
EU051633	2003	3.8	4.9
EU051634	2005	4.0	5.2
AGB56713	2007	3.7	4.8
HQ613402	2008	3.7	4.8
KM233035	2014	4.6	5.9
KJ660346	2014	4.1	5.3
KJ660348	2014	4.1	5.2

*(Data Sheet 1)*.

Systematic screening of 1012 FDA-approved drugs revealed amodiquine as the best candidate EBOV entry inhibitor (Madrid et al., 2013). We performed the docking analysis in order to identify a possible binding site for amodiaquine on EBOV GP1.

**Figure 7:**
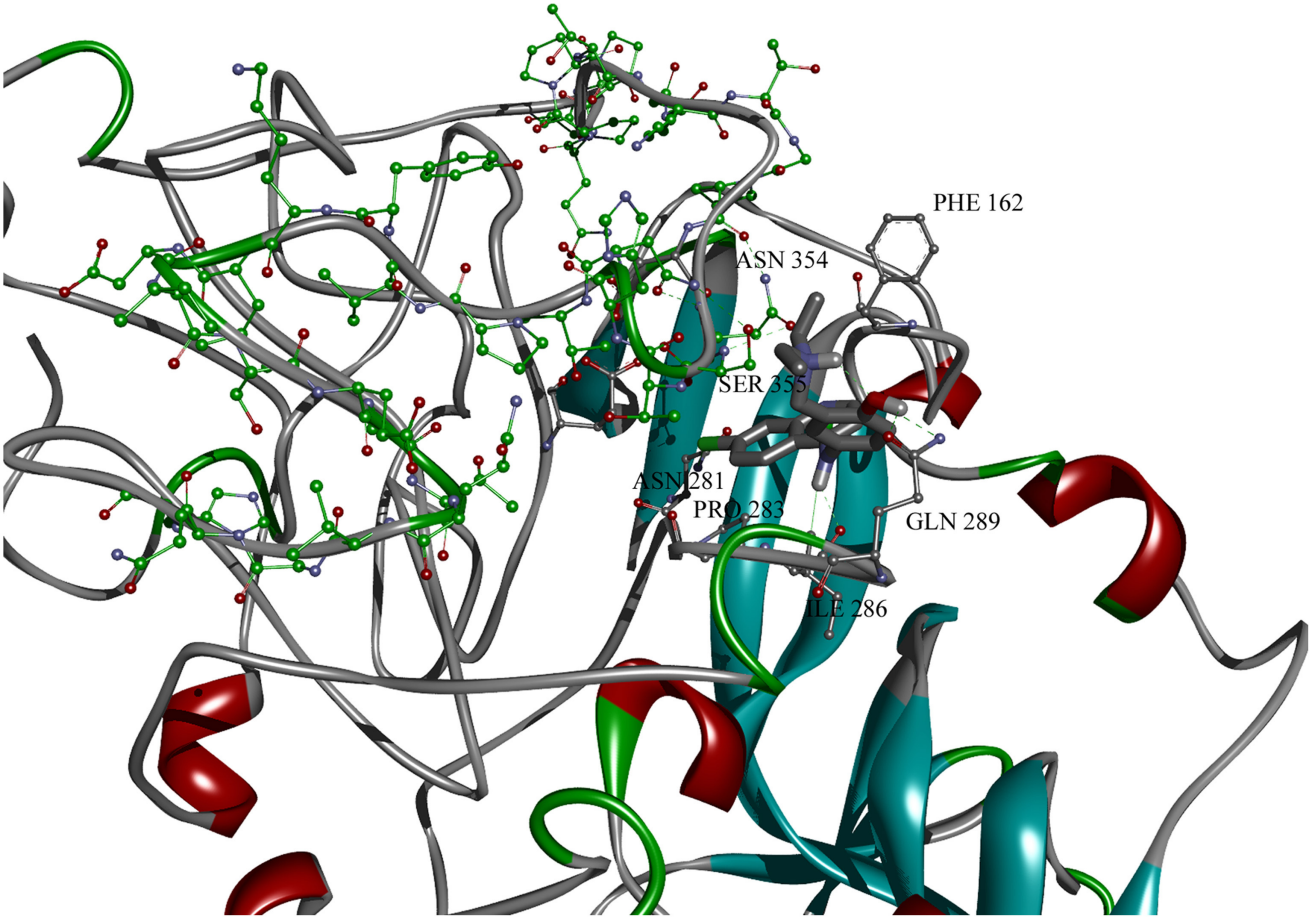
**Amodiquine docked into binding pocket of GP1 protein model with designated binding amino acid residues**. Residues from domain important for binding of antimalarics are colored in green.

After recognition of the protein domain important for binding of anti-malarials (Thr 341-His 375), the entire protein receptor conformational space was searched for binding pockets that include identified amino acids. As mentioned in Materials and Methods, the search was performed with and without emphasized hydrophilic interactions. During the conformational search, and only in the case of weighted hydrophilic interactions, conformations docked into binding pocket including recognized amino acids were found. The following amino acid residues were found to interact with amodiaquine: Pro 352, Asn 354, Ser 355, Gln 289, Ile 286, Pro 283, Asn 281, Glu 440, Asp 353, Phe 162. The gridbox was then moved and docking procedure was repeated, with the same parameters, but smaller grid box dimensions). The docked conformation of amodiaquine with lowest binding energy is presented on Figure 7. The stabilizing interactions - hydrophilic (with Asn 281, Ser 355, Asn 354, and Gln 289) and hydrophobic (CH-π with Pro 283, Ile 286, and possible aromatic with Phe 162) are thus formed between three loops, making it possible to stabilize protein conformation. On the other hand, there is no salt bridge found between protonated nitrogen and negatively charged amino acid residues in GP1, although there are candidates, such as Asp 353 or Glu 440, or other similar. Keeping in mind that the 3D coordinates of the discussed region are based only on primary structure, this can be only good guidance for insight into binding of antimalarials to GP1.

## Discussion

The EVD is a multisystemic disease which is characterized by hypotension, generalized fluid distribution balance, lymphopenia, coagulopathy, and hemorrhage. The potential role of endothelial cells in these various pathogenic processes is only partially understood. It was previously suggested that the EBOV GP is the main determinant of vascular cell injury and consequently it was proposed that the direct EBOV replication-induced structural damage of endothelial cells triggers the hemorrhagic diathesis (Baskerville et al., 1985; Geisbert et al., 1992). On the other hand, despite GP's preferential binding to endothelial cells, EBOV infection does not induce observable levels of apoptosis either directly or indirectly in endothelial cells neither *in vitro* nor *in vivo* (Geisbert et al., 2003). These results led to the conclusion that EBOV mediates disruption of endothelia via an indirect route rather than by direct cytolysis of endothelial cells (Geisbert et al., 2003). The EBOV GP is a potent activator of endothelial cells and also induces changes in the endothelial cell barrier function (Wahl-Jensen et al., 2005).

Proteins interacting with the GP that mediate endothelial disruption are not yet known. The present results suggest that EMILINs, expressed predominantly in the vasculature ECM and distributed throughout the blood vessel walls (for review see: Colombatti et al., 2012) are candidate binding proteins of GP1 binding. By applying the ISM analysis on the human proteome, we identified EMILINs as strong binding proteins of the protective antigen (PA) from *B. anthracis* and this *in silico* predicted interaction was experimentally confirmed *in vitro* (Fröhlich, 1968). We suggested that EMILINs, which are particularly expressed at and underneath the endothelium and in the vascular smooth muscle, could represent elective sites for *B. anthracis* tissue deposition. Consequently, *B. anthracis* could be concentrated at this site to play a direct role in the vascular lesions which are responsible for the prominent focal hemorrhages for human inhalational anthrax (Mayer et al., 2001) and for the injuries of aorta in similarly affected patients (Mayer et al., 2001; Holty et al., 2006). Similarly, EMILINs could function as elective sites for EBOV, thus facilitating infection of endothelial cells.

A prominent manifestation of EBOV infection is DIC, a syndrome characterized by systemic intravascular activation of coagulation generalized deposition of fibrin in the circulation (Sullivan et al., 2003). While EMILINs are known to play a role in coagulation, as a component of the vessel wall and/or a component of the thrombus (Sa et al., 2010; Sa and Hoover-Plow, 2011), these proteins could also be involved in DIC through interaction with GP1. EMILINs also play an important role in maintaining of ECM and vascular homeostasis (Colombatti et al., 2012). It was showed that EMILIN1 has the suppressive role in proteolytic degradation of ECM. This role is associated to both “structural” and “signaling mediated” functions of this protein (Danussi et al., 2012). Binding of GP to EMILIN1 could inhibit this protective function and allow ECM degradation. In general, it could be hypothesized that binding of GP to EMILINs contributes to EBOV infection and EVD pathogenesis by perturbation ECM and vascular homeostasis.

Computer-assisted scanning of the GP1 sequence indicated that the domain VIN_EBOV1_ (residues 341–375) was the most important domain for a strong signal at the frequency *F*_(0.338)_. According to the ISM concept, this domain would be involved in the direct interaction or immunological crossreactivity between EBOV GP1 and EMILINs. For this reason, VIN_EBOV1_ could be considered as a possible target for vaccination and treatment of EVD.

The ISM-based phylogenetic analysis (Figure 5) showed that GP1 of EBOV-2014 from Sierra Leone represented by virus KM233035 clusterized with EBOV from 1994-1996 outbreaks, and that the other two viruses KJ660346 and KJ660348 from Guinea 2014 are grouped with EBOV from more recent outbreaks. These results suggested different levels of interaction between these two groups of EBOV and EMILINs. The homology analysis of the highly variable region of GP1 (Rodriguez et al., 1999) showed that KM233035 was the most similar to EBOV from outbreaks 1994–1996 (Data Sheet 4).

It has been speculated that EBOV may rapidly accumulate mutations during passages in the human host, resulting in attenuation of the virus by the later stages of an outbreak (Rodriguez et al., 1999). Contrary to this assumption, it was shown that GP1 from EBOV-1995 in its highly variable region acquired mutation S345P, L346P, S390P, P398L, and S408G in this highly variable region that unexpectedly remained conserved after numerous human-to-human passages of the virus (Rodriguez et al., 1999). These five mutations, which were conserved as advantageous for the virus, appeared in viruses from outbreaks 1994 and 1996 (Data Sheet 4). Interestingly, the mutations S345P, L346P, S90P also appeared in viruses from later EBOV outbreaks; on the contrary, the mutations P398L and S408G were not present in viruses after 1996, with the only exception the viruses from the current outbreak 2014 in Sierra Leone. The GP1 from EBOV contains four of five mutations which are fixed in viruses from outbreaks 1994–1996 (Data Sheet 4) and these mutations are present in all 99 viruses from the outbreak 2014 represented with EBOV KM233035. Since the accidental acquisition and fixation of these four mutations within the highly variable region of GP1 is unlikely, we suggest that that EBOV from outbreaks in Sierra Leone is more connected with viruses from outbreaks 1994–1996 than with viruses from more recent outbreaks, as it was recently suggested (Gire et al., 2014).

Introduction of mutation S408G, which was absent from all the EBOV isolates after 1996, into EBOV KM233035 GP1 resulted in a significant increase of the amplitude at the frequency *F*_(0.338)_ (Figure 6). This suggests that acquisition of this mutation by EBOV during the current outbreak in West Africa could increase its interaction with ECMs expressing EMILINs, as already proposed by our work for the PA from *B. anthracis* and EMILINs (Doliana et al., 2008). By analogy, the increased binding of EBOV to EMILINs, expressed underneath the endothelium, could result in higher chances of airborne transmission of EBOV.

Recently published analysis of mutations in EBOV GP protein revealed 21 SNPs which affect binding of neutralizing antibodies preventing EBOV infection (Kugelman et al., 2015). Mutations P398L and S408G are among these SNPs which could influence prevention and therapy of EVD. These results additionally support our assumption about significance of these mutations which could help the virus to avoid the host immune response.

Vaccine against EBOV would be undoubtedly the most efficient way for controlling EBOV outbreaks. Two main obstacles in development of a vaccine are (i) binding of neutralizing antibodies by soluble GP1 (sGP1) which is in large amount secreted in the circulation during EBOV infection (antigenic subversion) (Mohan et al., 2012; Basler, 2013), and (ii) the antibody-dependent enhancement of EBOV infection (ADE) (Takada et al., 2007). Domain VIN_EMILIN_ which possibly is involved in GP1/EMILINs interaction is not present in sGP1 which is truncated form of GP1. Domain VIN_EMILIN_ also not contains epitopes required for ADE (Takada et al., 2007). Two monoclonal antibodies (Mabs) which are most effective in neutralization of EBOV are 6D8 Mab and 13F6 Mab (Kugelman et al., 2015). These Mabs are in the therapeutic cocktail MB-003 which represents one of three candidate therapeutic formulations for treatment of EVD. The epitope of 6D8 Mab is situated complete within the VIN-EMILIN peptide and the epitope of 13F6 Mab significantly overlaps this peptide (Wilson et al., 2000). For these reasons, the region of GP1 encompassing domain VIN_EMILIN_ could be considered as a candidate antigen for the EBOV vaccine.

Conventional time and money consuming approaches for drug development (>10 years; >2 billions $) does not meet the current urgent need for efficacious drugs for treatment of EBOLA disease. In this situation, an acceptable solution is repurposing existing drugs for treatment of EBOV infection, since currently approved drugs already have well-established safety and pharmacokinetic profiles in patients, as well as manufacturing and distribution networks. Recently, Madrid and co-workers performed a systematic screening of 1012 drugs approved in USA (Madrid et al., 2013) for their activity against EBOV. In this analysis 24 drugs as candidates for treatment of Ebola disease were selected. Among these drugs, the most effective are the antimalarials amodiaquone, chloroquine, and hydoxychloroquine. Performed docking analysis showed that amodiaqine possibly binds the pocket on GP1 which encompasses domain VIN_EBOV_. In this pocket, residues P352, D353, N354, and S355 directly interact with amodiaquine. Of note also is that not one of residues which are involved in binding of amodiaquine is within 21 SNPs affecting therapy of EVD (Kugelman et al., 2015). This suggests the region of EBOV GP1 encompassing domain VIN_EBOV_ represents a promising therapeutic target for treatment of EVD and anti-malarials as candidate drugs which are resistant to mutation of EBOV.

As a corollary, based on results of *in silico* analysis of the interaction between EBOV GP1 and host proteins, we hypothesizes (i) that EMILINs could participate in EBOV infection as elective sites for virus deposition on ECM, (ii) that mutations S3459, L346P, S390P, P398L, and S408G, are fixed as advantageous in the highly variable region of GP1 from EBOV isolated during the outbreaks 1994–1996, and (iii) that the region of EBOV GP1 encompassing domain VIN_EBOV_ and anti-malarials that bind to this region could be considered as a promising therapeutic target for candidate drugs for the treatment of EVD. These theoretical predictions, if experimentally confirmed, will help in better understanding the role of the endothelium in EBOV infection and could help in development of effective drugs for treatment of EVD.

## Author contributions

Conceived and designed the study, analysis and interpretation of data of the work, drafting the work, final approval of the version to be published: VV, AC, CPM, MS, DB. Developing the bioinformatics tools, analysis and interpretation of the data, drafting the work: SG, NV, VP. Molecular modeling: MS.

### Conflict of interest statement

The authors declare that the research was conducted in the absence of any commercial or financial relationships that could be construed as a potential conflict of interest.

## Supplementary material

Data Sheet 1 **GP1 from EBOV collected from 1976 to 2014**.

Data Sheet 2 **GP1 from EBOV collected in 2014**.

Data Sheet 3 **The multiple sequence alignment of GP1 from EBOV collected during outbreaks from 1994 to 2014**.

Data Sheet 4 **The multiple sequence alignment of the variable domain of GP1 from EBOV collected during outbreaks from 1994 to 2014**. Mutations S3459, L346P, S390P, P398L, and S408G are given in bold.
