# Supplementary material for: In silico analysis suggests interaction between Ebola virus and the extracellular matrix
Source: Front Microbiol. 2015 Feb 19;6:135. doi: 10.3389/fmicb.2015.00135 (PMC4333865; doi:10.3389/fmicb.2015.00135)
Supplement: Data Sheet 3 — The multiple sequence alignment of GP1 from EBOV collected during outbreaks from 1994 to 2014. [file DataSheet3.DOC]

Figure 1S

AHC70246-EBOV-1976 IPLGVIHNSTLQVSDVDKLVCRDKLSSTNQLRSVGLNLEGNGVATDVPSATKRWGFRSGV

AGB56749-EBOV-1977 IPLGVIHNSTLQVSDVDKLVCRDKLSSTNQLRSVGLNLEGNGVATDVPSATKRWGFRSGV

U77384-EBOV-1994 IPLGVIHNSTLQVSDVDKLVCRDKLSSTNQLRSVGLNLEGNGVATDVPSATKRWGFRSGV

AGB56767-EBOV-1996 IPLGVIHNSTLQVSDVDKLVCRDKLSSTNQLRSVGLNLEGNGVATDVPSATKRWGFRSGV

AGB56776-EBOV-1996 IPLGVIHNSTLQVSDVDKLVCRDKLSSTNQLRSVGLNLEGNGVATDVPSATKRWGFRSGV

AGB56821-EBOV-1995 IPLGVIHNSTLQVSDVDKLVCRDKLSSTNQLRSVGLNLEGNGVATDVPSATKRWGFRSGV

AGB56713-EBOV-2007 IPLGVIHNSTLQVSDVDKLVCRDKLSSTNQLRSVGLNLEGNGVATDVPSATKRWGFRSGV

HQ613402-EBOV-2008 IPLGVIHNSTLQVSDVDKLVCRDKLSSTNQLRSVGLNLEGNGVATDVPSATKRWGFRSGV

KJ660346-EBOV-2014 IPLGVIHNSTLQVSDVDKLVCRDKLSSTNQLRSVGLNLEGNGVATDVPSATKRWGFRSGV

KJ660348-EBOV-2014 IPLGVIHNSTLQVSDVDKLVCRDKLSSTNQLRSVGLNLEGNGVATDVPSATKRWGFRSGV

KM233035-EBOV-2014 IPLGVIHNSTLQVSDVDKLVCRDKLSSTNQLRSVGLNLEGNGVATDVPSVTKRWGFRSGV

EU051632-EBOV-2001 IPLGVIHNSTLQVSDVDKLVCRDKLSSTNQLRSVGLNLEGNGVATDVPSATKRWGFRSGV

EU051634-EBOV-2005 IPLGVIHNSTLQVSDVDKLVCRDKLSSTNQLRSVGLNLEGNGVATDVPSATKRWGFRSGV

EU051633-EBOV-2003 IPLGVIHNSTLQVSDVDKLVCRDKLSSTNQLRSVGLNLEGNGVATDVPSATKRWGFRSGV

EU051630-EBOV-2002 IPLGIIHNSTLQVSDVDKLVCRDKLSSTNQLRSVGLNLEGNGVATDVPSATKRWGFRSGV

AGB56830-EBOV-1996 IPLGVIHNSTLQVSDVDKLVCRDKLSSTNQLRSVGLNLEGNGVATDVPSATKRWGFRSGV

****:********************************************.**********

AHC70246-EBOV-1976 PPKVVNYEAGEWAENCYNLEIKKPDGSECLPAAPDGIRGFPRCRYVHKVSGTGPCAGDFA

AGB56749-EBOV-1977 PPKVVNYEAGEWAENCYNLEIKKPDGSECLPAAPDGIRGFPRCRYVHKVSGTGPCAGDFA

U77384-EBOV-1994 PPKVVNYEAGEWAENCYNLEIKKPDGSECLPAAPDGIRGFPRCRYVHKVSGTGPCAGDFA

AGB56767-EBOV-1996 PPKVVNYEAGEWAENCYNLEIKKPDGSECLPAAPDGIRGFPRCRYVHKVSGTGPCAGDFA

AGB56776-EBOV-1996 PPKVVNYEAGEWAENCYNLEIKKPDGSECLPAAPDGIRGFPRCRYVHKVSGTGPCAGDFA

AGB56821-EBOV-1995 PPKVVNYEAGEWAENCYNLEIKKPDGSECLPAAPDGIRGFPRCRYVHKVSGTGPCAGDFA

AGB56713-EBOV-2007 PPKVVNYEAGEWAENCYNLEIKKPDGSECLPAAPDGIRGFPRCRYVHKVSGTGPCAGDFA

HQ613402-EBOV-2008 PPKVVNYEAGEWAENCYNLEIKKPDGSECLPAAPDGIRGFPRCRYVHKVSGTGPCAGDFA

KJ660346-EBOV-2014 PPKVVNYEAGEWAENCYNLEIKKPDGSECLPAAPDGIRGFPRCRYVHKVSGTGPCAGDFA

KJ660348-EBOV-2014 PPKVVNYEAGEWAENCYNLEIKKPDGSECLPAAPDGIRGFPRCRYVHKVSGTGPCAGDFA

KM233035-EBOV-2014 PPKVVNYEAGEWAENCYNLEIKKPDGSECLPAAPDGIRGFPRCRYVHKVSGTGPCAGDFA

EU051632-EBOV-2001 PPKVVNYEAGEWAENCYNLEIKKPDGSECLPAAPDGIRGFPRCRYVHKVSGTGPCAGDFA

EU051634-EBOV-2005 PPKVVNYEAGEWAENCYNLEIKKPDGSECLPAAPDGIRGFPRCRYVHKVSGTGPCAGDFA

EU051633-EBOV-2003 PPKVVNYEAGEWAENCYNLEIKKPDGSECLPAAPDGIRGFPRCRYVHKVSGTGPCAGDFA

EU051630-EBOV-2002 PPKVVNYEAGEWAENCYNLEIKKPDGSECLPAAPDGIRGFPRCRYVHKVSGTGPCAGDFA

AGB56830-EBOV-1996 PPKVVNYEAGEWAENCYNLEIKKPDGSECLPAAPDGIRGFPRCRYVHKVSGTGPCAGDFA

************************************************************

AHC70246-EBOV-1976 FHKEGAFFLYDRLASTVIYRGTTFAEGVVAFLILPQAKKDFFSSHPLREPVNATEDPSSG

AGB56749-EBOV-1977 FHKEGAFFLYDRLASTVIYRGTTFAEGVVAFLILPQAKKDFFSSHPLREPVNATEDPSSG

U77384-EBOV-1994 FHKEGAFFLYDRLASTVIYRGTTFAEGVVAFLILPQAKKDFFSSHPLREPVNATEDPSSG

AGB56767-EBOV-1996 FHKEGAFFLYDRLASTVIYRGTTFAEGVVAFLILPQAKKDFFSSHPLREPVNATEDPSSG

AGB56776-EBOV-1996 FHKEGAFFLYDRLASTVIYRGTTFAEGVVAFLILPQAKKDFFSSHPLREPVNATEDPSSG

AGB56821-EBOV-1995 FHKEGAFFLYDRLASTVIYRGTTFAEGVVAFLILPQAKKDFFSSHPLREPVNATEDPSSG

AGB56713-EBOV-2007 FHKEGAFFLYDRLASTVIYRGTTFAEGVVAFLILPQAKKDFFSSHPLREPVNATEDPSSG

HQ613402-EBOV-2008 FHKEGAFFLYDRLASTVIYRGTTFAEGVVAFLILPQAKKDFFSSHPLREPVNATEDPSSG

KJ660346-EBOV-2014 FHKEGAFFLYDRLASTVIYRGTTFAEGVVAFLILPQAKKDFFSSHPLREPVNATEDPSSG

KJ660348-EBOV-2014 FHKEGAFFLYDRLASTVIYRGTTFAEGVVAFLILPQAKKDFFSSHPLREPVNATEDPSSG

KM233035-EBOV-2014 FHKEGAFFLYDRLASTVIYRGTTFAEGVVAFLILPQAKKDFFSSHPLREPVNATEDPSSG

EU051632-EBOV-2001 FHKEGAFFLYDRLASTVIYRGTTFAEGVVAFLILPQAKKDFFSSHPLREPVNATEDPSSG

EU051634-EBOV-2005 FHKEGAFFLYDRLASTVIYRGTTFAEGVVAFLILPQAKKDFFSSHPLREPVNATEDPSSG

EU051633-EBOV-2003 FHKEGAFFLYDRLASTVIYRGTTFAEGVVAFLILPQAKKDFFSSHPLREPVNATEDPSSG

EU051630-EBOV-2002 FHKEGAFFLYDRLASTVIYRGTTFAEGVVAFLILPQAKKDFFSSHPLREPVNATEDPSSG

AGB56830-EBOV-1996 FHKEGAFFLYDRLASTVIYRGTTFAEGVVAFLILPQAKKDFFSSHPLREPVNATEDPSSG

************************************************************

AHC70246-EBOV-1976 YYSTTIRYQATGFGTNETEYLFEVDNLTYVQLESRFTPQFLLQLNETIYTSGKRSNTTGK

AGB56749-EBOV-1977 YYSTTIRYQATGFGTNETEYLFEVDNLTYVQLESRFTPQFLLQLNETIYTSGKRSNTTGK

U77384-EBOV-1994 YYSTTIRYQATGFGTNETEYLFEVDNLTYVQLESRFTPQFLLQLNETRYTSGKRSNTTGK

AGB56767-EBOV-1996 YYSTTIRYQATGFGTNETEYLFEVDNLTYVQLESRFTPQFLLQLNETIYTSGKRSNTTGK

AGB56776-EBOV-1996 YYSTTIKYQATGFGTNETEYLFEVDNLTYVQLESRFTPQFLLQLNETIYTSGKRSNTTGK

AGB56821-EBOV-1995 YYSTTIRYQATGFGTNETEYLFEVDNLTYVQLESRFTPQFLLQLNETIYTSGKRSNTTGK

AGB56713-EBOV-2007 YYSTTIRYQATGFGTNETEYLFEVDNLTYVQLESRFTPQFLLQLNETIYASGKRSNTTGK

HQ613402-EBOV-2008 YYSTTIRYQATGFGTNETEYLFEVDNLTYVQLESRFTPQFLLQLNETIYASGKRSNTTGK

KJ660346-EBOV-2014 YYSTTIRYQATGFGTNETEYLFEVDNLTYVQLESRFTPQFLLQLNETIYASGKRSNTTGK

KJ660348-EBOV-2014 YYSTTIRYQATGFGTNETEYLFEVDNLTYVQLESRFTPQFLLQLNETIYASGKRSNTTGK

KM233035-EBOV-2014 YYSTTIRYQATGFGTNETEYLFEVDNLTYVQLESRFTPQFLLQLNETIYASGKRSNTTGK

EU051632-EBOV-2001 YYSTTIRYQATGFGTNETEYLFEVDNLTYVQLESRFTPQFLLQLNETIYASGKRSNTTGK

EU051634-EBOV-2005 YYSTTIRYQATGFGTNETEYLFEVDNLTYVQLESRFTPQFLLQLNETIYASGKRSNTTGK

EU051633-EBOV-2003 YYSTTIRYQATGFGTNETEYLFEVDNLTYVQLESRFTPQFLLQLNETIYASGKRSNTTGK

EU051630-EBOV-2002 YYSTTIRYQATGFGTNETEYLFEVDNLTYVQLESRFTPQFLLQLNETIYASGKRSNTTGK

AGB56830-EBOV-1996 YYSTTIRYQATGFGTNETEYLFEVDNLTYVQLESRFTPQFLLQLNETIYASGKRSNTTGK

******:**************************************** *:**********

AHC70246-EBOV-1976 LIWKVNPEIDTTIGEWAFWETKKNLTRKIRSEELSFTVVSNGAKNISGQSPARTSSDPGT

AGB56749-EBOV-1977 LIWKVNPEIDTTIGEWAFWETKKNLTRKIRSEELSFTVVSNGAKNISGQSPARTSSDPGT

U77384-EBOV-1994 LIWKVNPEIDTTIGEWAFWETKKNLTRKIRSEELSFTAVSNRAKNISGQSPARTSSDPGT

AGB56767-EBOV-1996 LIWKVNPEIDTTIGEWAFWETKKNLTRKIRSEELSFTAVSNRAKNISGQSPARTSSDPGT

AGB56776-EBOV-1996 LIWKVNPEIDTTIGEWAFWETKKNLTRKIRSEELSFTAVSNRAKNISGQSPARTSSDPGT

AGB56821-EBOV-1995 LIWKVNPEIDTTIGEWAFWETKKNLTRKIRSEELSFTAVSNRAKNISGQSPARTSSDPGT

AGB56713-EBOV-2007 LIWKVNPEIDTTIGEWAFWETKKNLTRKIRSEELSFTAVSNGAKNLSGQSPARTSSDPKT

HQ613402-EBOV-2008 LIWKVNPEIDTTIGEWAFWETKKNLTRKIRSEELSFTAVSNGAKNLSGQSPARTSSDPKT

KJ660346-EBOV-2014 LIWKVNPEIDTTIGEWAFWETKKNLTRKIRSEELSFTAVSNGPKNISGQSPARTSSDPET

KJ660348-EBOV-2014 LIWKVNPEIDTTIGEWAFRETKKNLTRKIRSEELSFTAVSNGPKNISGQSPARTSSDPET

KM233035-EBOV-2014 LIWKVNPEIDTTIGEWAFWETKKNLTRKIRSEELSFTAVSNGPKNISGQSPARTSSDPET

EU051632-EBOV-2001 LIWKVNPEIDATIGEWAFWETKKTSLGKIRSEELSFTAVSNGAKDISGQSPARTSSDPET

EU051634-EBOV-2005 LIWKVNPEIDTTIGEWAFWETKKTSLGKIRSEELSFTAVSNGAKDIGGQSPARTSSDPET

EU051633-EBOV-2003 LIWKVNPEIDTTIGEWAFWETKKTSLGKIRSEELSFTAVSNGAKDISGQSPARTSSDPET

EU051630-EBOV-2002 LIWKVNPEIDTTIGEWAFWETKKTSLGKIRSEELSFTAVSNGAKDISGQSPARTSSDPET

AGB56830-EBOV-1996 LIWKVNPEIDTTIGEWAFWETKKNLTRKIRSEELSFTAVSNGAKDISGQSPARTSSDPET

**********:******* ****. **********.*** .*::.*********** *

AHC70246-EBOV-1976 NTTTEDHKIMASENSSAMVQVHSQGREAAVSHLTTLATISTSPQSLTTKPGPDNSTHNTP

AGB56749-EBOV-1977 NTTTEDHKIMASENSSAMVQVHSQGREAAVSHLTTLATISTSPQSLTTKPGPDNSTHNTP

U77384-EBOV-1994 NTTTEDHKIMASENSSAMVQVHSQGREAAVSHLTTLATISTSLRPPITKPGPDNSTHNTP

AGB56767-EBOV-1996 NTTTEDHKIMASENSSAMVQVHSQGREAAVSHLTTLATISTSLQPPTTKPGPDNSTHNTP

AGB56776-EBOV-1996 NTTTEDHKIMASENSSAMVQVHSQGREAAVSHLTTPATISTSLQPPTTKPGPDNSTHNTP

AGB56821-EBOV-1995 NTTTEDHKIMASENSSAMVQVHSQGREAAVSHLTTLATISTSPQPPTTKPGPDNSTHNTP

AGB56713-EBOV-2007 NTTTEDHKIVASENSSAMVQVHSQGREAAVSHLTTLATISTSPQPPTTKPGPDNSTYNTP

HQ613402-EBOV-2008 NTTTEDHKIVASENSSAMVQVHSQGREAAVSHLTTLATISTSPQPPTTKPGPDNSTYNTP

KJ660346-EBOV-2014 NTTNEDHKIMASENSSAMVQVHSQGRKAAVSHLTTLATISTSPQSLTTKPGPDNSTHNTP

KJ660348-EBOV-2014 NTTNEDHKIMASENSSAMVQVHSQGRKAAVSHLTTLATISTSPQSLTTKPGPDNSTHNTP

KM233035-EBOV-2014 NTTNEDHKIMASENSSAMVQVHSQGRKAAVSHLTTLATISTSPQPPTTKTGPDNSTHNTP

EU051632-EBOV-2001 YTTTEDHKIMASENSSAMVQVHNQGREAAVSHLITLATISTSPQSPTTKPGQDNSTHNTP

EU051634-EBOV-2005 YTTTEDHKIMASENSSTMVQVHNQGREAAVSHLITLATISTSPQSPTTKPGQDNSTHNTP

EU051633-EBOV-2003 YTTTEDHKIMASENSSAMVQVHNQGREAAVSHLITLATISTSPQSPTTKPGQDNSTHNTP

EU051630-EBOV-2002 YTTTGDHKIMASENSSAMVQVHNQGREAAVSHLITFATISTSPQSPTTKPGQDNSRANTP

AGB56830-EBOV-1996 YTTTEDHKIMASENSSAMVQVHNQGREAAVSHLITLATISTSPQSPTTKPGQDNSTHNTP

**. ****:******:*****.***:****** * ****** :. **.* *** ***

AHC70246-EBOV-1976 VYKLDISEATQVEQHHRRTDNDSTASDTPSATTAAGPPKAENTNTSKSTDFLDPATTTSP

AGB56749-EBOV-1977 VYKLDISEATQVEQHHRRTDNDSTASDTPSATTAAGPPKAENTNTSKSTDFLDPATTTSP

U77384-EBOV-1994 VYKLDISEATQVEQHHRRTDNASTTSDTPPATTAAGPLKAENTNTSKGTDLLDPATTTSP

AGB56767-EBOV-1996 VYKLDISEATQVEQHHRRTDNASTTSDTPPATTAAGPLKAENTNTSKGTDLLDPATTTSP

AGB56776-EBOV-1996 VYKLDISEATQVEQHHRRTDNASTTSDTPPATTAAGPLKAENTNTSKGTDLLDPATTTSP

AGB56821-EBOV-1995 VYKLDISEATQVEQHHRRTDNDSTASDTPPATTAAGPLKAENTNTSKGTDLLDPATTTSP

AGB56713-EBOV-2007 VYKLDTSEATQVEQHHRRTDNDSTASDTPPATTAAGHPKAENTNTSKSADSLDPATTTSP

HQ613402-EBOV-2008 VYKLDTSEATQVEQHHRRTDNDSTASDTPPATTAAGHPKAENTNTSKSADSLDPATTTSP

KJ660346-EBOV-2014 VYKLDISEATQVGQHHRRADNDSTASDTPPATTAAGPLKAENTNTSKSADSLDLATTTSP

KJ660348-EBOV-2014 VYKLDISEATQVGQHHRRADNDSTASDTPPATTAAGPLKAENTNTSKSADSLDLATTTSP

KM233035-EBOV-2014 VYKLDISEATQVGQHHRRADNDSTASDTPPATTAAGPLKAENTNTSKSADSLDLATTTSP

EU051632-EBOV-2001 VYKLDISEATQVEQHHRRTDNDSTASDTPPATTAAGPPKAENINTSKSADSLDPATTTSP

EU051634-EBOV-2005 VYKLDISEATQVEQHHRRTDNDSTASDTPPATTAAGPPKAENINTSKSADSLDPATTTSP

EU051633-EBOV-2003 VYKLDISEATQVEQHHRRTDNDSTASDTPPATTAAGPPKAENINTSKSADSLDPATTTSP

EU051630-EBOV-2002 VYKLDISEATQVEQHHRRTDNDSTASDTPPATTAAGPPKAENINTSKSADSLDPATTTSP

AGB56830-EBOV-1996 VYKLDISEATQVEQHHRRTDNDSTASDTPPATTAAGPPKAENINTSKSADSLDPATTTSP

***** ****** *****:** **:****.****** **** ****.:* ** ******

AHC70246-EBOV-1976 QNHSETAGNNNTHHQDTGEESASSGKLGLITNTIAGVAGLITGGRRTRR

AGB56749-EBOV-1977 QNHSETAGNNNTHHQDTGEESASSGKLGLITNTIAGVAGLITGGRRTRR

U77384-EBOV-1994 QNHSETAGNNNTHHQDTGEESASSGKLGLITNTIAGVAGLITGGRRTRR

AGB56767-EBOV-1996 QNHSETAGNNNTHHQDTGEESASSGKLGLITNTIAGVAGLITGGRRTRR

AGB56776-EBOV-1996 QNHSETAGNNNTHHQDTGEESTSSGKLGLITNTIAGVAGLITGGRRTRR

AGB56821-EBOV-1995 QNHSETAGNNNTHHQDTGEESASSGKLGLITNTIAGVAGLITGGRRARR

AGB56713-EBOV-2007 PNHSETAGNNNTHHQDTGEESASSGKLGLITNTIAGVAGLITGGRRTRR

HQ613402-EBOV-2008 PNHSETAGNNNTHHQDTGEESASSGKLGLITNTIAGVAGLITGGRRTRR

KJ660346-EBOV-2014 QNYSETAGNNNTHHQDTGEESASSGKLGLITNTIAGVAGLITGGRRTRR

KJ660348-EBOV-2014 QNYSETAGNNNTHHQDTGEESASSGKLGLITNTIAGVAGLITGGRRTRR

KM233035-EBOV-2014 QNYSETAGNNNTHHQDTGEESASSGKLGLITNTIAGVAGLITGGRRTRR

EU051632-EBOV-2001 QNYSETAGNNNTHHQDTGEESAGSGKLGLITNTIAGVAGLITGGRRTRR

EU051634-EBOV-2005 QNYSETAGNNNTHHQDTGEESAGSGKLGLITNTIAGVAGLITGGRRTRR

EU051633-EBOV-2003 QNYSETAGNNNTHHQDTGEESAGSGKLGLIANTIAGVAGLITGGRRTRR

EU051630-EBOV-2002 QNYSETAGNNNTHHQDTGEESAGSGKLGLITNTIAGVAGLITGGRRTRR

AGB56830-EBOV-1996 QNYSETAGNNNTHHQDTGEESAGSGKLGLIANTIAGVAGLITGGRRTRR

*:******************:.*******:***************:**
