# Supplementary material for: In silico analysis suggests interaction between Ebola virus and the extracellular matrix
Source: Front Microbiol. 2015 Feb 19;6:135. doi: 10.3389/fmicb.2015.00135 (PMC4333865; doi:10.3389/fmicb.2015.00135)
Supplement: Data Sheet 4 — The multiple sequence alignment of the variable domain of GP1 from EBOV collected during outbreaks from 1994 to 2014. Mutations S3459, L346P, S390P, P398L, and S408G are given in bold. [file DataSheet4.DOC]

Figure 2S

AHC70246-EBOV-1976 VSHLTTLATISTSPQSLTTKPGPDNSTHNTPVYKLDISEATQVEQHHRRTDNDSTASDTPSATTAAGPPKAENTNTSKS

AGB56749-EBOV-1977 VSHLTTLATISTSPQSLTTKPGPDNSTHNTPVYKLDISEATQVEQHHRRTDNDSTASDTPSATTAAGPPKAENTNTSKS

U77384-EBOV-1994 VSHLTTLATISTSLR**PP**ITKPGPDNSTHNTPVYKLDISEATQVEQHHRRTDNASTTSDTP**P**ATTAAGP**L**KAENTNTSK**G**

AGB56821-EBOV-1995 VSHLTTLATISTSPQ**PP**TTKPGPDNSTHNTPVYKLDISEATQVEQHHRRTDNASTTSDTP**P**ATTAAGP**L**KAENTNTSK**G**

AGB56767-EBOV-1996 VSHLTTLATISTSLQ**PP**TTKPGPDNSTHNTPVYKLDISEATQVEQHHRRTDNASTTSDTP**P**ATTAAGP**L**KAENTNTSK**G**

AGB56776-EBOV-1996 VSHLTTPATISTSLQ**PP**TTKPGPDNSTHNTPVYKLDISEATQVEQHHRRTDNASTTSDTP**P**ATTAAGP**L**KAENTNTSK**G**

AGB56830-EBOV-1996 VSHLITLATISTSPQS**P**TTKPGQDNSTHNTPVYKLDISEATQVEQHHRRTDNDSTASDTP**P**ATTAAGPPKAENINTSKS

EU051632-EBOV-2001 VSHLITLATISTSPQS**P**TTKPGQDNSTHNTPVYKLDISEATQVEQHHRRTDNDSTASDTP**P**ATTAAGPPKAENINTSKS

EU051630-EBOV-2002 VSHLITFATISTSPQS**P**TTKPGQDNSRANTPVYKLDISEATQVEQHHRRTDNDSTASDTP**P**ATTAAGPPKAENINTSKS

EU051633-EBOV-2003 VSHLITLATISTSPQS**P**TTKPGQDNSTHNTPVYKLDISEATQVEQHHRRTDNDSTASDTP**P**ATTAAGPPKAENINTSKS

EU051634-EBOV-2005 VSHLITLATISTSPQS**P**TTKPGQDNSTHNTPVYKLDISEATQVEQHHRRTDNDSTASDTP**P**ATTAAGPPKAENINTSKS

AGB56713-EBOV-2007 VSHLTTLATISTSPQ**PP**TTKPGPDNSTYNTPVYKLDTSEATQVEQHHRRTDNDSTASDTP**P**ATTAAGHPKAENTNTSKS

HQ613402-EBOV-2008 VSHLTTLATISTSPQ**PP**TTKPGPDNSTYNTPVYKLDTSEATQVEQHHRRTDNDSTASDTP**P**ATTAAGHPKAENTNTSKS

KJ660346-EBOV-2014 VSHLTTLATISTSPQSLTTKPGPDNSTHNTPVYKLDISEATQVGQHHRRADNDSTASDTP**P**ATTAAGP**L**KAENTNTSKS

KJ660348-EBOV-2014 VSHLTTLATISTSPQSLTTKPGPDNSTHNTPVYKLDISEATQVGQHHRRADNDSTASDTP**P**ATTAAGP**L**KAENTNTSKS

KM233035-EBOV-2014 VSHLTTLATISTSPQ**PP**TTKTGPDNSTHNTPVYKLDISEATQVGQHHRRADNDSTASDTP**P**ATTAAGP**L**KAENTNTSKS

**** * ****** :. **.* *** ******** ****** *****:** **:****.****** **** ****.

AGB56821-EBOV-1995 VSHLTTLATISTSPQ**PP**TTKPGPDNSTHNTPVYKLDISEATQVEQHHRRTDNASTTSDTP**P**ATTAAGP**L**KAENTNTSK**G**

KM233035-EBOV-2014 VSHLTTLATISTSPQ**PP**TTKTGPDNSTHNTPVYKLDISEATQVGQHHRRADNDSTASDTP**P**ATTAAGP**L**KAENTNTSKS

******************************************* *****:****************************

KJ660348-EBOV-2014 VSHLTTLATISTSPQSLTTKPGPDNSTHNTPVYKLDISEATQVGQHHRRADNDSTASDTP**P**ATTAAGP**L**KAENTNTSKS

KM233035-EBOV-2014 VSHLTTLATISTSPQ**PP**TTKTGPDNSTHNTPVYKLDISEATQVGQHHRRADNDSTASDTP**P**ATTAAGP**L**KAENTNTSKS

***************. **************************************************************
